# Bis(oxazolines) based on glycopyranosides – steric, configurational and conformational influences on stereoselectivity

**DOI:** 10.3762/bjoc.6.23

**Published:** 2010-03-04

**Authors:** Tobias Minuth, Mike M K Boysen

**Affiliations:** 1 Institute of Organic Chemistry, Gottfried-Wilhelm-Leibniz University of Hannover, Schneiderberg 1B, D-30167 Hannover, Germany

**Keywords:** asymmetric synthesis, carbohydrates, copper, cyclopropanation, ligand design

## Abstract

In previous studies we found that the asymmetric induction of bis(oxazolines) based on D-glucosamine strongly depended on the steric demand of the 3-*O*-substituents. To further probe the impact of the 3-position of the pyranose scaffold, we prepared 3-epimerised and 3-defunctionalised versions of these ligands as well as a 3-*O*-formyl derivative. Application of these new ligands in asymmetric cyclopropanation revealed strong steric and configurational effects of position 3 on asymmetric induction, further dramatic effects of the pyranose conformation were also observed.

## Introduction

The design and optimisation of chiral ligands for metal catalysed transformations is of crucial importance for stereoselective synthesis and is therefore an active field of research. In this context, carbohydrates are interesting, even if comparatively rarely used as starting materials for the preparation of new chiral ligand structures. Today, 30 years after the first reports on carbohydrate-based ligands [1–4], the potential of saccharide compounds in this area is more and more appreciated [5–12].

Chiral bis(oxazolines) (Box) are very efficient ligands for many asymmetric transformations [13–14]. Even though N-acylated derivatives of D-glucosamine easily form bicyclic carbohydrate oxazolines, until recently only a few examples of mono(oxazoline) ligands [15–17] and the corresponding bis(oxazolines) [18] based on this monosaccharide have appeared in the literature. In the course of our work we have introduced new glucosamine-derived bis(oxazolines) **2a**–**c** with uniform protective groups on all oxygen functions [19–21] and derivatives **3a**–**f** with cyclic 4,6-*O*-benzylidene protection as well as various other 3-*O*-substituents that differ in steric demand and electronic nature [20–21].

**Scheme 1:**
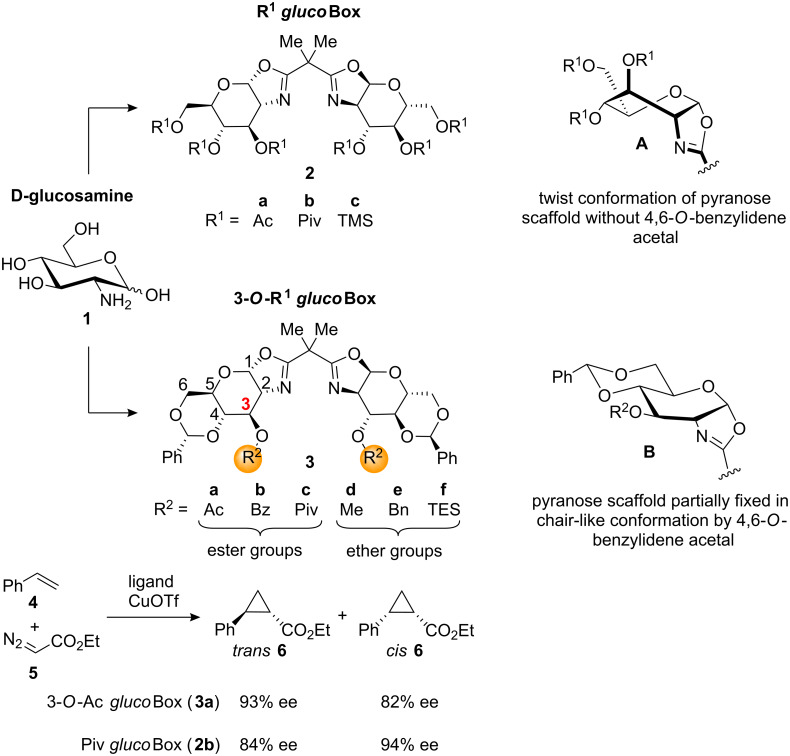
New glucosamine-based bis(oxazoline) ligands with their pyranose conformation and application in asymmetric cyclopropanation.

These ligands were subsequently employed in the asymmetric cyclopropanation [22–23] of styrene (**4**) with ethyl diazoacetate (**5**). Our results revealed a strong dependence of the enantioselectivity on both the steric bulk and electronic nature of the *O*-substituents in ligands **2a**–**c** and **3a**–**f**. Furthermore, the conformation of the pyranose scaffold – a twist conformation for ligands **2a**–**c** without 4,6-*O*-benzylidene protection (Scheme 1, conformer **A**) and a partially chair-like conformation for ligands **3a**–**f** (Scheme 1, conformer **B**) fixed by the annulated 4,6-*O*-benzylidene group – has a direct impact on the enantioselectivity of the reaction. For ligands **3a**–**f** with cyclic protection, a decrease in the bulk of the 3-*O*-residues led to an improvement in stereoselectivity, while the opposite trend was observed for counterparts **2a**–**c** with acyclic 4,6-*O*-protection. Moreover, ester modified ligands **2a**, **2b** and **3a**–**c** led to higher stereoselectivity than the corresponding ether-modified compounds **2c** and **3d**–**f**. The best results were obtained with 3-*O*-Ac *gluco*Box **3a** that combines a small 3-*O*-acyl residue with cyclic 4,6-*O*-protection, and with bulky ligand Piv *gluco*Box (**2b**) without any cyclic protection. These findings are summarised in Scheme 1.

**Figure 1:**
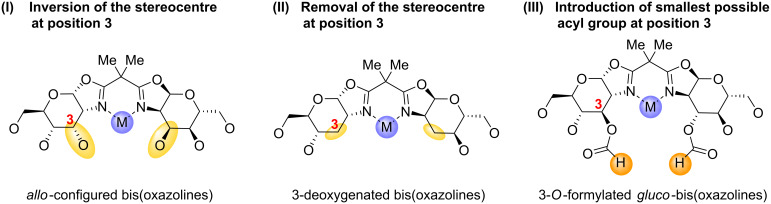
Planned modifications at pyranose position 3 of carbohydrate bis(oxazolines).

Because of the strong impact of the pyranose position 3 in ligands **3a**–**f** on the stereoselectivity, we became interested in elucidating the influence of the stereochemistry at this position by both 3-epimerisation and 3-defunctionalisation. Inversion of the configuration at position 3 to give an *allo*-configured ligand scaffold, will bring the 3-*O*-substituent into a *syn*-relationship with the oxazoline nitrogen atom and therefore into very close proximity to a coordinated metal centre (Figure 1, **I**). Deoxygenation of the 3-postion on the other hand will lead to a ligand with comparably little steric shielding of metal centres coordinated by the oxazoline nitrogen atoms (Figure 1, **II**). As the stereoselectivity of the model reaction for ligands **3a**–**f** improved with decreasing steric demand of the 3-*O*-substituent and since the best results were obtained with acyl-modified ligands, we also set out to prepare a corresponding ligand with a formyl group as the smallest possible acyl residue at the 3-*O*-position. In this paper we describe the synthesis of new 3-epimerised and 3-deoxygenated carbohydrate bis(oxazolines), the preparation of a 3-*O*-formate analogue of ligands **3** as well as the testing of these new ligands in stereoselective cyclopropanation.

## Results and Discussion

**Scheme 2:**
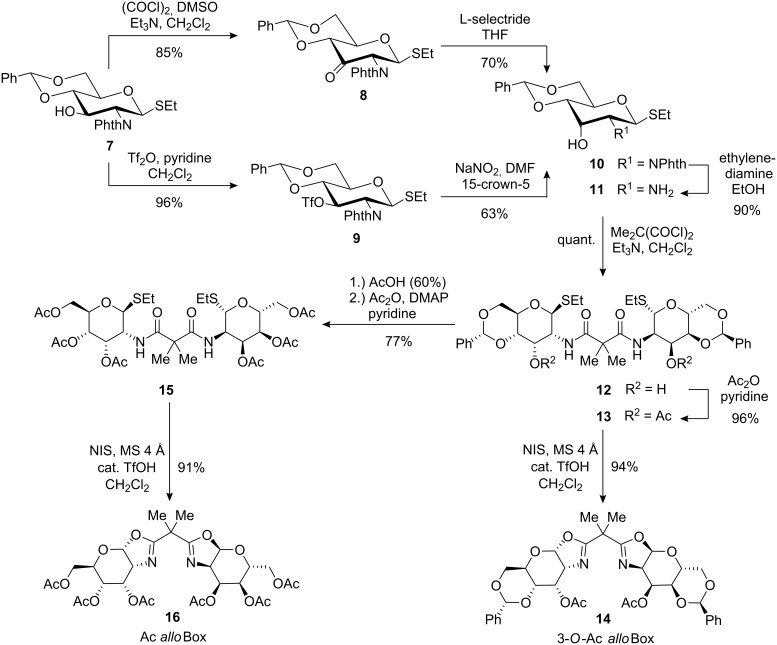
Synthesis of *allo*-configured bis(oxazolines) 3-*O*-Ac *allo*Box (**14**) and Ac *allo*Box (**16**) from thioglucoside **7** as the key intermediate.

The synthesis of all new ligands started from the known thioglucoside **7** [24] which was also employed as key intermediate for the preparation of ligands **3a**–**f** [20–21] and is accessible from D-glucosamine in 5 steps and 57% overall yield. To prepare an *allo*-configured precursor for ligand synthesis, we decided first to use a previously described epimerisation sequence for **7** featuring Swern oxidation and subsequent reduction with sodium borohydride [25]. In our hands this method led to an inseparable product mixture in the second step however, on switching to L-selectride for the stereoselective reduction [26], the allosamine derived thioglycoside **10** was obtained in good overall yield. For an alternative route, **7** was transformed into the 3-*O*-triflate **9** and then subjected to nucleophilic displacement with sodium nitrite in the presence of 15-crown-5 [27–28] to afford **10** in similar yields as the oxidation-reduction sequence (Scheme 2). After deprotection of the phthalimide (phthN) [29], the free amine **11** was transformed into the 4,6-*O*-benzylidene protected ligand by our standard protocol for the preparation of carbohydrate bis(oxazoline) ligands [20–21]: Formation of bis(amide) **12** with dimethylmalonyl chloride, 3-*O*-acetylation and subsequent activation of the thioethyl moieties of **13** with NIS [30] for the double cyclisation step, led to benzylidene protected ligand 3-*O*-Ac *allo*Box **14** in excellent yield. As noted previously, the presence of a 4,6-*O*-benzylidene group has a pronounced influence on the conformation adopted by the pyranose scaffold in *gluco*-configured ligands (Scheme 1, conformers **A** and **B**), which in turn has a direct influence on the stereoselectivity in the model reaction. In order to ascertain if a similar conformative effect is also in operation for *allo*-configured bis(oxazolines), we prepared ligand Ac *allo*Box **16** with acyclic 4,6-*O*-protection by the removal of the benzylidene groups from **12** under acidic conditions and per-*O*-acetylation in a one-pot reaction followed by NIS-mediated cyclisation of resulting bis(amide) **15**.

**Scheme 3:**
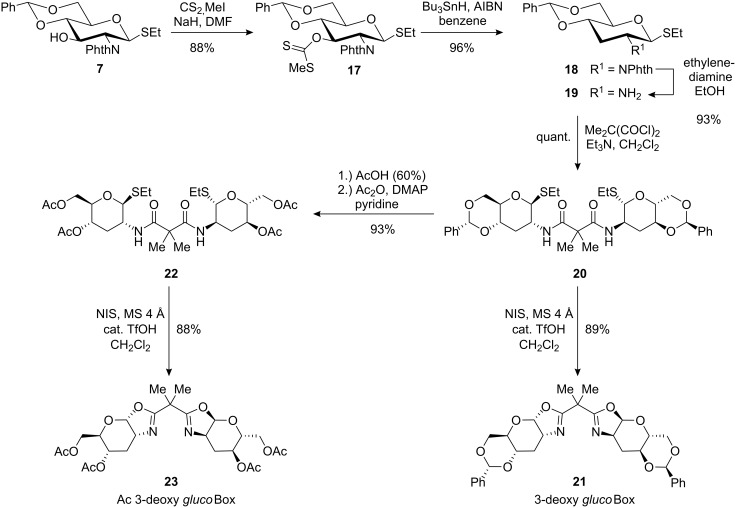
Preparation of ligands 3-deoxy *gluco*Box (**21**) and Ac 3-deoxy *gluco*Box (**23**) from key intermediate **7**.

For the preparation of 3-deoxygenated ligands, we planned a defunctionalisation of the key intermediate **7**. Surprisingly, a thorough search of the literature revealed only one example of the 3-deoxygenation of a glucosamine-derived thioglycoside, reported by Herdewijn et al. in 2006 [31]. Because the Barton–McCombie deoxygenation [32] failed on their *N*-Troc protected thio aminoglucoside under various conditions, Herdewijn et al. used a sequence via a 3-iodide derivative. To avoid the rather complicated preparation of a 3-iodo derivative, we tried the Barton–McCombie reaction on our phthalimido protected precursor **7** (Scheme 3). Introduction of the 3-xanthogenate with carbon disulfide and methyl iodide yielded **17**, which was cleanly deoxygenated in high yield by tributyltin hydride under standard conditions [32–33]. From the resulting compound **18**, the ligands 3-deoxy *gluco*Box **21** with benzylidene groups and Ac 3-deoxy *gluco*Box **23** with acyclic 4,6-*O*-protection were prepared in high overall yields (Scheme 3).

**Scheme 4:**
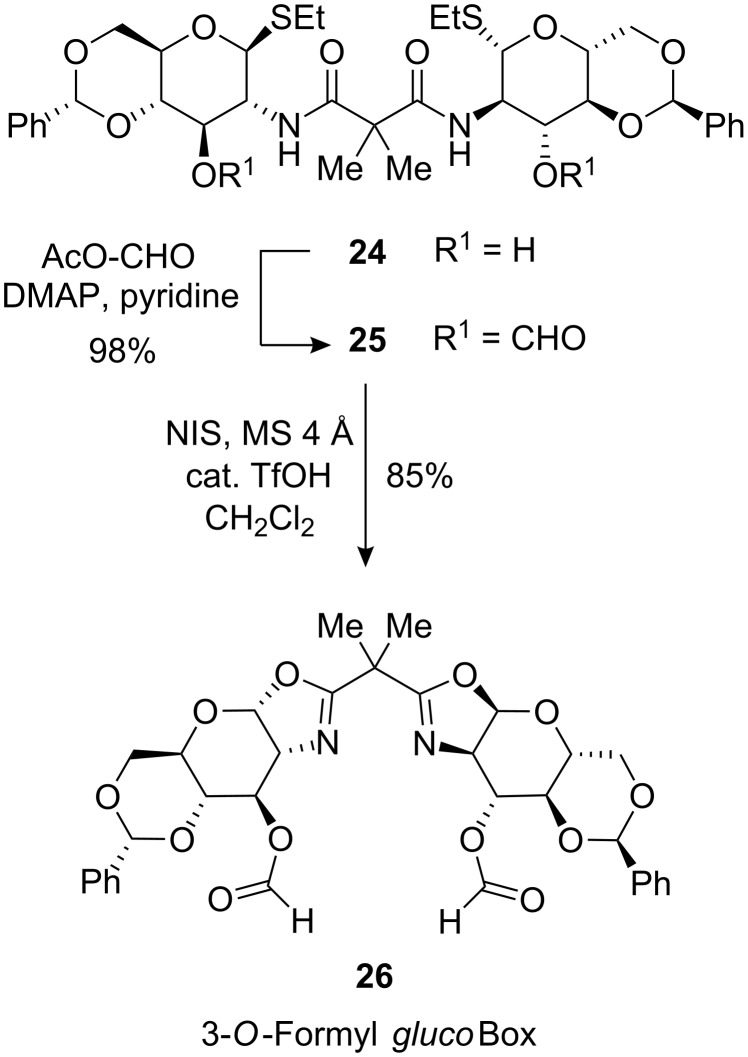
Preparation of ligand 3-*O*-Formyl *gluco*Box (**26**) from bis(amide) **24**.

The 3-*O*-formate analogue of *gluco*-configured ligands **3** was obtained by treatment of bis(amide) **24** [20–21] with formyl acetate [34] to yield **25** which was then cyclised to the desired ligand **26** with NIS (Scheme 4).

The five new ligands **14**, **16**, **21**, **23** and **26** were now employed in the copper(I) catalysed asymmetric cyclopropanation of styrene (**4**) with diazoacetate (**5**) under known conditions [19,21–22] (Table 1). All ligands led to formation of the products *trans* **6** and *cis* **6** in good to excellent yields and the *trans*/*cis* ratio was in the typical range (around 70:30) obtained with bis(oxazoline) ligands [22]. However, the enantioselectivities differed dramatically for the new ligands and revealed once again the strong influence of position 3 and the pyranose conformation on the efficiency of the asymmetric induction. The best results were obtained with 3-*O*-formyl *gluco*Box **26** which gave *trans*** 6** and *cis* **6** in 95% ee and 94% ee respectively (Table 1, entry 5).

**Table 1:** Cyclopropanations with *allo*-configured ligands **14** and **16**, 3-deoxygenated ligands **21** and **23** and 3-*O*-formylated, *gluco*-configured ligand **26**.

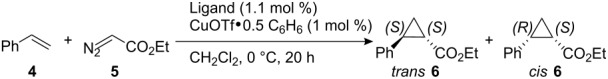					
Entry	Ligand	Yield [%]^a^	*trans*/*cis*^b^	ee *trans* [%]^b^	ee *cis* [%]^b^
1	3-*O*-Ac *allo*Box (**14**)	75	66:34	rac.	rac.
2	Ac *allo*Box (**16**)	79	70:30	71	87
3	3-deoxy *gluco*Box (**21**)	86	69:31	rac.	rac.
4	Ac 3-deoxy *gluco*Box (**23**)	75	74:26	78	72
5	3-*O*-formyl *gluco*Box (**26**)	95	71:29	95	94

^a^Isolated yield after chromatography. ^b^Determined by GC on a chiral stationary phase.

**Figure 2:**
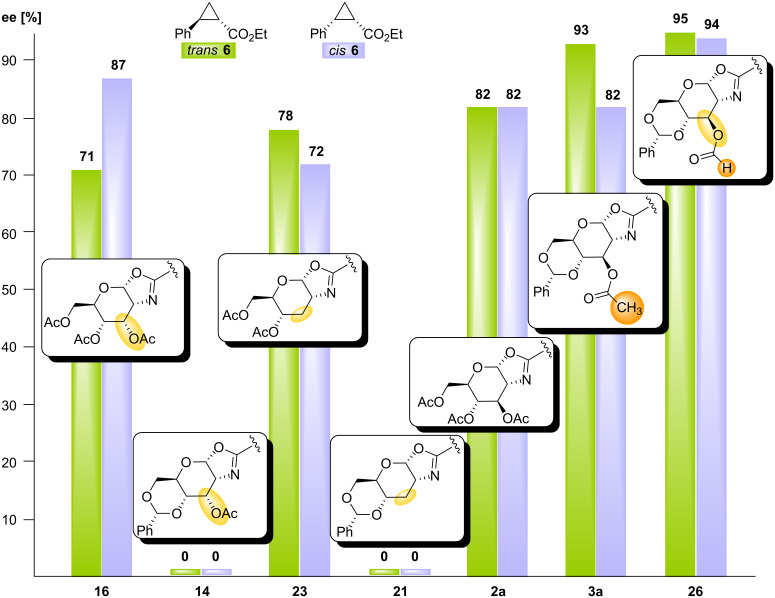
Impact of structural ligand modifications on the stereoselectivity of cyclopropanations.

Figure 2 gives a summary of the results obtained with the new ligands as well as a comparison with the previously reported ligands **2a** and **3a**. Both, benzylidene-protected ligands 3-*O*-Ac *allo*Box **14** and 3-deoxy *gluco*Box **21** gave only racemic products while their counterparts Ac *allo*Box **16** and Ac 3-Deoxy *gluco*Box **23** lacking cyclic 4,6-*O*-protection led to substantial asymmetric induction. This demonstrates that the dramatic conformational effect of the pyranose scaffold on stereoselectivity, which was first observed for *gluco*-configured ligands **2** and **3**, is also in operation in *allo*- and 3-deoxy *gluco*-ligands. However, while benzylidene protection in 3-*O*-Ac *gluco*Box **3a** led to improved asymmetric induction in comparison to ligand Ac *gluco*Box **2a** lacking cyclic protection, the opposite was observed for the *allo*- and 3-deoxy-ligands. The strong influence of the configuration of pyranose position 3 on stereoselectivity becomes apparent by a comparison of ligand 3-*O*-Ac *gluco*Box **3a** to its 3-epimerised and 3-defunctionalised counterparts **14** and **21**: Both modifications, inversion of the configuration in *allo*-ligand **14** and 3-defunctionalisation in **21** result in a complete loss of stereoselectivity in the model reaction, whilst 3-*O*-Ac *gluco*Box **3a** provides the products in 93% ee and 82% ee respectively. Finally, *gluco*-configured ligand **26** with a 3-*O*-formyl residue led to higher stereoselectivities (95% ee and 94% ee for *trans* **6** and *cis ***6** respectively) than 3-*O*-acetylated ligand **3a**. This confirms the trend we initially observed for *gluco*-configured ligands. A decrease in steric bulk of 3-*O*-acyl substituents results in improved asymmetric induction of the ligand in the cyclopropanation reaction: ee for 3-*O*-Piv **3c** < 3-*O*-Bz **3b** < 3-*O*-Ac **3a** < 3-*O*-Formyl **26**. Thus, of all carbohydrate-derived bis(oxazolines) prepared by us, ligand **26** led to the best enantioselectivities for cyclopropanes *trans* **6** and *cis*** 6**.

## Conclusion

We have prepared new derivatives of *gluco*-configured bis(oxazoline) ligands **2** and **3** with 3-epimerisation or 3-defunctionalisation in the pyranose scaffold. Application in stereoselective cyclopropanation as a model reaction highlighted the strong impact of modifications at the pyranose position 3 on the asymmetric induction exerted by carbohydrate-based ligands. Furthermore, the previously observed conformational effect of cyclic 4,6-*O*-benzylidene protection on stereoselectivity is also in operation in the new derivatives. Introduction of a 3-*O*-formate in *gluco*Box ligands led to improved stereoselectivities compared to the corresponding 3-*O*-acetate. This underlines our previous findings that the best results for *gluco*-configured ligands are obtained with small acyl-based 3-*O*-substitutents. The observed steric, configurational and conformational effects are as yet not fully understood and investigations to elucidate their origins are currently under way.

## Supporting Information

Supporting information contains full experimental details for the preparation of all new ligands and general conditions for cyclopropanations using *gluco*Box ligands and copper(I) triflate.

File 1 Experimental details.
