# Supplementary material for: Bis(oxazolines) based on glycopyranosides – steric, configurational and conformational influences on stereoselectivity
Source: Beilstein J Org Chem. 2010 Mar 4;6:23. doi: 10.3762/bjoc.6.23 (PMC2874406; doi:10.3762/bjoc.6.23)
Supplement: File 1 — Experimental details. [file Beilstein_J_Org_Chem-06-23-s001.pdf]

Supporting Information

for

Bis(oxazolines) based on glycopyranosides – steric, configurational and conformational influences on stereoselectivity

Tobias Minuth and Mike M. K. Boysen*

Address: Institute of Organic Chemistry, Gottfried-Wilhelm Leibniz University of Hannover, Schneiderberg 1B, D-30167 Hannover, Germany

Email: Mike M. K. Boysen - mike.boysen@oci.uni-hannover.de

* Corresponding author

General methods

Dry solvents were obtained by distillation over appropriate drying reagents under a nitrogen atmosphere (CH_2Cl_2 was distilled from calcium hydride), or purchased in dried form from commercial sources (DMF and benzene from Acros, abs. ethanol from Fisher Scientific). All reactions involving reagents sensitive to air and moisture were carried out under a nitrogen atmosphere (glove box and/or Schlenk techniques). Reactions were monitored by TLC on 60 F254 aluminium plates (Merck) with detection by UV light and/or charring with 10% sulfuric acid in ethanol or a mixture of cerium(IV) sulfate and molybdophosphoric acid in 8% sulfuric acid. Flash chromatography was performed on Merck silica (grain size 40–63 μm). NMR spectra were recorded on an AVS 400 instrument (Bruker) at 400 MHz (^{1}H) or at 100 MHz (^{13}C) respectively. Deuterated chloroform was used as solvent and spectra were calibrated against the residual solvent peaks ($CHCl_3$: 7.24 ppm for ^{1}H and 77 ppm for ^{13}C). Chemical shifts δ are given in ppm, coupling constants J are given in Hz. Electrospray mass (ESI) spectra were recorded on a Micromass LCT device (Waters). Optical rotations were recorded on a Perkin-Elmer 451 instrument under following standard conditions: Room temperature, wavelength 589.3 nm (sodium D line), cell length 1 dm, solvent and sample concentration (in 10 mg/ml) are given with the individual experiment.

Ethyl 4,6-*O*-benzylidene-2-deoxy-2-phthalimido-1-thio-*β*-D-glucopyran-3-ulose (8)

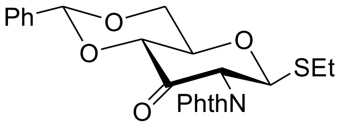

Oxalyl chloride (2.65 g, 1.79 mL, 20.84 mmol) was dissolved in dry CH_2Cl_2 (60 mL) at −78 °C. Dimethylsulfoxide (2.04 g, 1.85 mL, 26.05 mmol) was added slowly and the reaction mixture stirred for 15 min at −78 °C. Thioglucoside **7** (2.30 g, 5.21 mmol) dissolved in dry CH_2Cl_2 (10 mL) was then added slowly and the reaction mixture stirred for further 20 min at −78 °C. Et_3N (5.00 mL) was added and the mixture allowed to warm to room temperature. The mixture was diluted with CH_2Cl_2, washed successively with saturated aqueous $NaHCO_3$ solution and brine, and dried over Na_2SO_4. The solvent was removed under reduced pressure and the residue purified by flash chromatography on silica gel (eluent: PE/EtOAc 4:1) to yield **8** (1.95 g, 4.43 mmol, 85%) as a colourless foam.

***R*f** 0.76 (2:1 PE/EtOAc); **^{1}H NMR** ($CDCl_3$, 400 MHz): δ = 7.84–7.87 (m, 2H, Phth), 7.70–7.75 (m, 2H, Phth), 7.47–7.50 (m, 2H, Ph), 7.32–7.36 (m, 3H, Ph), 5.64 (d, 1H, $J_{1,2}$ = 10.2 Hz, H-1), 5.58 (s, 1H, CHPh), 4.96 (d, 1H, $J_{1,2}$ = 10.2 Hz, H-2), 4.52 (dd, 1H, $J_{5,6}$ = 4.7 Hz, $J_{6,6'}$ = 10.2 Hz, H-6), 4.42 (d, 1H, $J_{4,5}$ = 9.9 Hz, H-4), 4.01 (ddd ≈ td, 1H, $J_{4,5} \approx J_{5,6'}$ = 9.9 Hz, $J_{5,6}$ = 4.7 Hz, H-5), 3.92 (dd ≈ t, 1H, $J_{5,6'}$ = 9.9, $J_{6,6'}$ = 10.2 Hz, H-6′), 2.68–2.78 (m, 2H, SCH_2CH_3), 1.22 (t, 3H, J = 7.5 Hz, SCH_2CH_3) ppm; **^{13}C NMR** ($CDCl_3$, 100 MHz,): δ = 191.4 (C, C3), 167.04 (C, NCO), 136.1 (C, arom.), 134.4, 134.3 (CH, arom.), 131.5, 131.5 (C, arom.), 129.3, 128.2, 126.3, 123.8, 123.7, (CH, arom.), 101.9 (CH, PhCH), 84.0 (CH, C-1), 82.0 (CH, C-4), 71.2 (CH, C-5), 68.9 (CH_2, C-6), 59.1 (CH, C-2), 24.7 (CH_2, SCH_2CH_3), 14.8 (CH_3, SCH_2CH_3) ppm; **HRMS** (ESI): calculated for $C_{23}H_{25}N_2O_6S$ 457.1428, found 457.1439 $[M + NH_4]^+$.

Ethyl 4,6-*O*-benzylidene-2-deoxy-2-phthalimido-1-thio-3-trifluoromethane-sulfonyl-*β*-D-glucopyranoside (9)

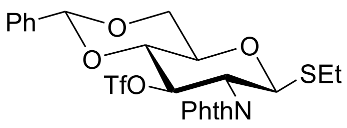

Thioglucoside **7** (1.00 g, 2.27 mmol) was dissolved in dry CH_2Cl_2 (15 mL), cooled to −20 °C and pyridine (1.54 g, 1.58 mL, 19.52 mmol) was added. Trifluoromethanesulfonic anhydride (1.28 g, 0.75 mL, 4.54 mmol) was dissolved in dry CH_2Cl_2 (5 mL) and added slowly to the reaction mixture at −20 °C. The mixture was allowed to warm up to 10 °C and diluted with CH_2Cl_2 and HCl (1 M). The mixture was washed successively with saturated aqueous $NaHCO_3$ solution and brine. The organic layer was dried over Na_2SO_4 and the solvent removed under reduced pressure. The residue was purified by flash chromatography on silica gel (eluent: PE/EtOAc 3:1) to yield **9** (1.22 g, 2.13 mmol, 96%) as a colourless foam.

R_f 0.78 (3:1 PE/EtOAc); **^{1}H NMR** ($CDCl_3$, 400 MHz): δ = 7.87–7.92 (m, 2H, Phth), 7.73–7.78 (m, 2H, Phth), 7.45–7.48 (m, 2H, Ph), 7.33–7.39 (m, 3H, Ph), 5.76 (dd, 1H, $J_{2,3}$ = 9.5 Hz, $J_{3,4}$ = 9.2 Hz, H-3), 5.61 (s, 1H, CHPh), 5.46 (d, 1H, $J_{1,2}$ = 10.2 Hz, H-1), 4.58 (t ≈ dd, 1H, $J_{1,2}$ = 10.2 Hz, $J_{2,3}$ = 9.5 Hz, H-2), 4.45 (dd, 1H, $J_{5,6}$ = 4.4 Hz, $J_{6,6'}$ = 9.9 Hz, H-6), 3.92 (dd ≈ t, 1H, $J_{3,4}$ = 9.2 Hz, $J_{4,5}$ = 9.9 Hz, H-4), 3.84 (dd ≈ t, 1H, $J_{5,6'}$ ≈ $J_{6,6'}$ = 9.9 Hz, H-6´), 3.77 (ddd ≈ td, 1H, $J_{4,5}$ ≈ $J_{5,6'}$ = 9.9 Hz, $J_{5,6}$ = 4.4 Hz, H-5), 2.59–2.73 (m, 2H, S$CH_2$$CH_3$), 1.18 (t, 3H, *J* = 7.5 Hz, $SCH_2$$CH_3$) ppm; **^{13}C NMR** ($CDCl_3$, 100 MHz): *δ* = 167.9, 166.6 (C, NCO), 136.1 (C, arom.), 134.6 (CH, arom.), 131.3, 131.0 (C, arom.), 129.1, 128.2, 125.8, 124.0, 123.6 (CH, arom.), 101.3 (CH, PhCH), 82.9 (CH, C3), 81.8 (CH, C-1), 78.5 (CH, C-4), 70.2 (CH, C-5), 68.3 (CH_2, C-6), 53.6 (CH, C-2), 24.3 (CH_2, S$CH_2$$CH_3$), 14.7 ($CH_3$, $SCH_2$$CH_3$) ppm; **HRMS** (ESI): calculated for $C_{24}H_{22}F_3NNaO_8S_2$ 596.0631, found 596.0681 $[M + Na]^+$.

Ethyl 4,6-*O*-benzylidene-2-deoxy-2-phthalimido-1-thio-*β*-D-allopyranoside (10)

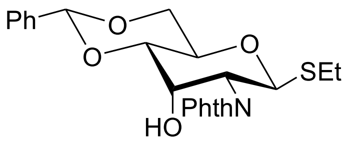

From ulose 8: Compound **8** (480 mg, 1.09 mmol) was dissolved in dry THF (20 mL) and cooled to −78 °C. A solution of L-selectride® (1 M, THF, 1.31 mL) was added slowly and the reaction mixture stirred for 2 h at −78 °C. The reaction was quenched with water, the solvent removed under reduced pressure and the residue twice co-evaporated with toluene. The residue was purified by flash chromatography on silica gel (eluent: PE/EtOAc 3:1) to yield **10** (330 mg, 760 µmol, 70%) as a colourless powder.

From Triflate 9: Compound **9** (200 mg, 350 µmol) was dissolved in dry DMF (10 mL). Successively, 15-crown-5 (230 mg, 210 µL, 1.05 mmol) and $NaNO_2$ (70 mg, 1.05 mmol) were added. The reaction mixture was heated to 50 °C and stirred for 24 h. The solvent was removed under reduced pressure and the residue co-evaporated twice with toluene. The residue was purified by flash chromatography on silica gel (eluent: PE/EtOAc 3:1) to yield **10** (96 mg, 220 µmol, 63%) as a colourless powder.

$\boldsymbol{R_f}$ 0.51 (3:1 PE/EtOAc); $[\alpha]_D^{20}$ = −58.1 (*c* = 1.15, $CHCl_3$); **^{1}H NMR** ($CDCl_3$, 400 MHz): δ = 7.83–7.88 (m, 2H, Phth), 7.70–7.75 (m, 2H, Phth), 7.45–7.48 (m, 2H, Ph), 7.33–7.37 (m, 3H, Ph), 6.01 (d, 1H, $J_{1,2}$ = 10.5 Hz, H-1), 5.59 (s, 1H, CHPh), 4.40–4.44 (m, 3H, 2-H, 3-H, H-6), 4.23 (ddd ≈ td, 1H, $J_{4,5}$ = 9.5 Hz, $J_{5,6'}$ = 10.2 Hz, $J_{5,6}$ = 5.1 Hz, H-5), 3.79 (dd ≈ t, 1H, $J_{5,6'} \approx J_{6,6'}$ = 10.2 Hz, H-6′), 3.75 (dd, 1H, $J_{3,4}$ = 2.3 Hz, $J_{4,5}$ = 9.5 Hz, H-4), 3.17 (br. s, 1 H, 3-OH), 2.62–2.74 (m, 2H, SCH_2CH_3), 1.21 (t, 3H, *J* = 7.5 Hz, SCH_2CH_3) ppm; **^{13}C NMR** ($CDCl_3$, 100 MHz): δ = 168.8 167.7 (C, NCO), 136.9 (C, arom.), 134.3, 134.2 (CH, arom.), 131.8, 131.2 (C, arom.), 129.2, 128.3, 126.2

123.7, 123.6 (CH, arom.), 101.9 (CH, PhCH), 79.1 (CH, C-1), 78.9 (CH, C-4), 69.3 (CH, C-3), 69.0 (CH_2, C-6), 66.4 (CH, C-5), 55.2 (CH, C-2), 24.7 (CH_2, S*C*H_2CH_3), 14.9 (CH_3, SCH_2*C*H_3) ppm; **HRMS** (ESI): calculated for $C_{23}H_{23}NO_6SNa$ 464.1138, found 464.1141 $[M + Na]^+$.

Ethyl 2-amino-4,6-*O*-benzylidene-2-deoxy-1-thio-*β*-D-allopyranoside (11)

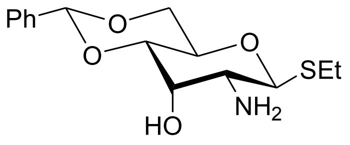

N-Protected allosamine **10** (500 mg, 1.13 mmol) and ethylenediamine (2.80 mL, 4.07 g, 67.80 mmol) were dissolved in abs. ethanol (50 mL) and the reaction mixture refluxed for 16 h (TLC: EtOAc). The solvent was removed under reduced pressure and the residue co-evaporated twice with toluene. The residue was purified by flash chromatography on silica gel (eluent: CH_2Cl_2/MeOH 20:1) to yield **11** (320 mg, 1.02 mmol, 90%) as a colourless powder.

***R*$_f$** 0.21 (20:1 CH_2Cl_2/MeOH); **$[\alpha]_D^{20}$** = −54.2 (*c* = 1.06, $CHCl_3$); **^{1}H NMR** ($CDCl_3$, 400 MHz): δ = 7.44–7.49 (m, 2H, Ph), 7.32–7.39 (m, 3H, Ph), 5.53 (s, 1H, CHPh), 4.59 (d, 1H, $J_{1,2}$ = 10.2 Hz, H-1), 4.34 (dd, 1H, $J_{5,6}$ = 5.1 Hz, $J_{6,6'}$ = 10.2 Hz, H-6), 4.22 (dd ≈ t, 1H, $J_{2,3}$ = 2.7 Hz, $J_{3,4}$ = 2.3 Hz, H-3), 3.96 (ddd ≈ td, 1H, $J_{4,5} \approx J_{5,6'}$ = 9.9 Hz, $J_{5,6}$ = 5.1 Hz, H-5), 3.70 (dd ≈ t, 1H, $J_{5,6} \approx J_{6,6'}$ = 10.2 Hz, H-6′), 3.52 (dd, 1H, $J_{3,4}$ = 2.3 Hz, $J_{4,5}$ = 9.9 Hz, H-4), 2.74 (dd, 1H, $J_{1,2}$ = 10.2 Hz, $J_{2,3}$ = 2.7 Hz, H-2), 2.60–2.72 (m, 2H, SC*H*$_2$CH$_3$), 1.28 (t, 3H, *J* = 7.5 Hz, SCH$_2$C*H*$_3$) ppm; **^{13}C NMR** ($CDCl_3$, 100 MHz): δ = 137.1 (C, Ph), 129.2, 128.3, 126.1 (CH, Ph), 101.8 (CH, PhCH), 85.9 (CH, C-1), 79.6 (CH, C-4), 69.1 (CH_2, C-6), 68.2 (CH, C-3), 66.0 (CH, C-5), 54.1 (CH, C-2), 24.5 (CH_2, S*C*H_2CH_3), 15.2 (CH_3, SCH_2*C*H_3) ppm; **HRMS** (ESI): calculated for $C_{15}H_{21}NO_4SNa$ 334.1083, found 334.1079 $[M + Na]^+$.

***N,N'*-Bis(ethyl 2-amino-4,6-*O*-benzylidene-2-deoxy-1-thio-*β*-D-allopyranosid-2-yl)dimethylmalonamide (12)**

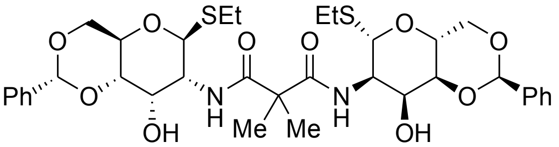

Allosamine **11** (200 mg, 640 µmol) was dissolved in dry CH_2Cl_2 (40 mL) under a nitrogen atmosphere and cooled to 0 °C. Successively Et_3N (180 µL, 130 g, 1.28 mmol) and dimethylmalonyl dichloride (50 µL, 60 mg, 320 µmol) were added (TLC: EtOAc). After approximately 2 h the solvent was removed under reduced pressure and the residue purified by flash chromatography on silica gel (eluent: PE/EtOAc 1:1) to yield **12** (230 mg, 320 µmol, quant.) as colourless crystals.

***R*f** 0.51 (1:1 PE/EtOAc); **$[\alpha]_D^{20}$** = −90.3 (*c* = 0.80, $CHCl_3$); **^{1}H NMR** ($CDCl_3$, 400 MHz): δ = 7.42–7.44 (m, 4H, Ph), 7.33–7.35 (m, 6H, Ph), 6.86 (d, 2H, $J_{2,NH}$ = 8.8 Hz, NH), 5.55 (s, 2H, CHPh), 4.75 (d, 2H, $J_{1,2}$ = 9.9 Hz, H-1), 4.35 (dd, 2H, $J_{5,6}$ = 5.1 Hz, $J_{6,6'}$ = 10.2 Hz, H-6), 4.20-4.26 (m, 4H, 2-H, H-3), 3.95 (ddd ≈ td, 2H, $J_{4,5} \approx J_{5,6'}$ = 9.5 Hz, $J_{5,6}$ = 5.1 Hz, H-5), 3.72 (dd ≈ t, 2H, $J_{5,6'} \approx J_{6,6'}$ = 10.2 Hz, H-6´), 3.62 (dd, 2H, $J_{3,4}$ = 2.0 Hz, $J_{4,5}$ = 9.5 Hz, H-4), 2.73 (br. s, 2H, 3-OH), 2.68–2.71 (m, 4H, $SC\mathit{H}_2CH_3$), 1.48 [s, 6H, $(CH_3)_2C$], 1.25 (t, 6H, *J* = 7.5 Hz, $SCH_2C\mathit{H}_3$) ppm; **^{13}C NMR** ($CDCl_3$, 100 MHz): δ = 172.9 (C, CONH), 136.8 (C, Ph), 129.2, 128.3, 126.1 (CH, Ph), 101.6 (CH, PhCH), 82.1 (CH, C-1), 78.6 (CH, C-4), 68.9 (CH_2, C-6), 67.9 (CH, C-3), 66.2 (CH, C-5), 51.1 (CH, C-2), 49.9 [C, $(CH_3)_2\mathit{C}$], 23.9 [CH_3, $(\mathit{C}H_3)_2C$], 23.8 (CH_2, $S\mathit{C}H_2CH_3$), 14.8 (CH_3, $SCH_2\mathit{C}H_3$) ppm; **HRMS** (ESI): calculated for $C_{35}H_{47}N_2O_{10}S_2$ 719.2667, found 719.2661 $[M + H]^+$.

***N,N'*-Bis(ethyl 3-*O*-acetyl-4,6-*O*-benzylidene-2-deoxy-1-thio-*β*-D-allopyranosid-2-yl)dimethylmalonamide (13)**

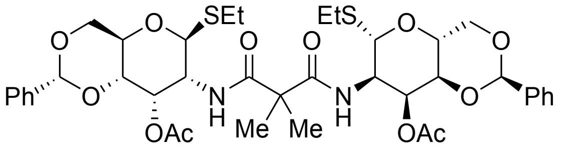

Bis(amide) **12** (190 mg, 260 μmol) was dissolved in pyridine (20 mL) and acetic anhydride (250 μL, 270 mg, 2.64 mmol) added slowly. The reaction mixture was stirred for 16 h at room temperature (TLC: PE/EtOAc 1:1). The solvent was removed under reduced pressure and the residue co-evaporated twice with toluene. The residue was purified by flash chromatography on silica gel (eluent: PE/EtOAc 2:1) to yield **13** (200 mg, 250 μmol, 96%) as a colourless foam.

$\boldsymbol{R}_\mathbf{f}$ 0.43 (1:1 PE/EtOAc); $[\alpha]_D^{20}$ = −81.2 (c = 0.93, $CHCl_3$); **^{1}H NMR** ($CDCl_3$, 400 MHz): δ = 7.36–7.39 (m, 4H, Ph), 7.30–7.33 (m, 6H, Ph), 6.70 (d, 2H, $J_{2,NH}$ = 8.8 Hz, NH), 5.57 (dd ≈ t, 2H, $J_{2,3} \approx J_{3,4}$ = 2.7 Hz, H-3), 5.53 (s, 2H, CHPh), 4.73 (d, 2H, $J_{1,2}$ = 10.5 Hz, H-1), 4.35 (dd, 2H, $J_{5,6}$ = 4.7 Hz, $J_{6,6'}$ = 10.2 Hz, H-6), 4.30 (ddd ≈ td, 2H, $J_{1,2}$ = 10.5 Hz, $J_{2,3}$ = 3.0 Hz, $J_{2,NH}$ = 8.8 Hz, H-2), 3.90 (ddd ≈ td, 2H, $J_{4,5} \approx J_{5,6'}$ = 9.5 Hz, $J_{5,6}$ = 4.7 Hz, H-5), 3.74 (dd, 2H, $J_{5,6'} \approx J_{6,6'}$ = 10.2 Hz, H-6′), 3.73 (dd, 2H, $J_{3,4}$ = 2.7 Hz, $J_{4,5}$ = 9.5 Hz, H-4), 2.69–2.76 (m, 4H, $SC\mathit{H}_2CH_3$), 1.40 [s, 6H, $(CH_3)_2C$], 2.17 (s, 6H, CH_3CO), 1.26 (t, 6H, J = 7.5 Hz, $SCH_2C\mathit{H}_3$) ppm; **^{13}C NMR** ($CDCl_3$, 100 MHz): δ = 172.7 (C, CONH), 169.5 (C, $CH_3\mathit{C}O$), 136.8 (C, Ph), 129.0, 128.2, 125.9 (CH, Ph), 101.3 (CH, PhCH), 82.3 (CH, C-1), 76.9 (CH, C-4), 68.97 (CH, C-3), 68.94 (CH_2, C-6), 67.4 (CH, C-5), 50.2 (CH, C-2), 49.5 [C, $(CH_3)_2\mathit{C}$], 23.9 [CH_3, $(\mathit{C}H_3)_2C$], 23.8 (CH_2, $S\mathit{C}H_2CH_3$), 20.8 (CH_3, $\mathit{C}H_3CO$), 14.9 (CH_3, $SCH_2\mathit{C}H_3$) ppm; **HRMS** (ESI): calculated for $C_{39}H_{50}N_2O_{12}NaS_2$ 825.2703, found 825.2689 $[M + Na]^+$.

3-*O*-Ac *allo*Box (14)

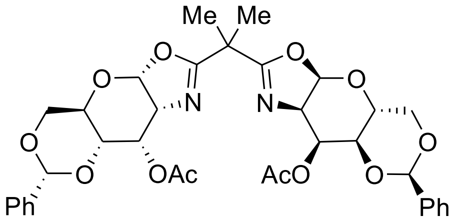

A mixture of bis(amide) **13** (130 mg, 160 μmol) and MS 4 Å (130 mg) in dry CH_2CH_2 (5 mL) was stirred for 1 h under a nitrogen atmosphere. NIS (88 mg, 390 μmol) was added, and the mixture cooled to −30 °C. Then, TfOH (1.8 μL, 20 μmol) was added, and the mixture was stirred for 1 h at −30 °C. The reaction was quenched by addition of Et_3N (100 μL), filtered through Celite®, diluted with CH_2Cl_2, washed successively with saturated aqueous $NaHCO_3$ solution and aqueous sodium thiosulfate solution (3 M), and dried over Na_2SO_4. The solvent was removed under reduced pressure and the residue purified by flash chromatography on silica gel (eluent: EtOAc) to yield **14** (100 mg, 150 μmol, 94%) as a colourless solid.

***R*$_f$** 0.05 (1:1 PE/EtOAc); **[*α*]$_D^{20}$** = +192.8 (*c* = 0.76, $CHCl_3$); **^{1}H NMR** ($CDCl_3$, 400 MHz): *δ* = 7.38–7.41 (m, 4H, Ph), 7.32–7.34 (m, 6H, Ph), 5.83 (d, 2H, $J_{1,2}$ = 6.8 Hz, H-1), 5.64 (dd, 2H, $J_{2,3}$ = 5.8 Hz, $J_{3,4}$ = 2.3 Hz, H-3), 4.38 (dd, 2H, $J_{5,6}$ = 5.1 Hz, $J_{6,6'}$ = 10.5 Hz, H-6), 5.56 (s, 2H, CHPh), 4.28 (ddd ≈ td, 2H, $J_{4,5}$ = $J_{5,6'}$ = 9.5 Hz, $J_{5,6}$ = 5.1 Hz, H-5), 4.14 (dd, 2H, $J_{1,2}$ = 6.8 Hz, $J_{2,3}$ = 5.8 Hz, H-2), 3.69 (dd, 2H, $J_{3,4}$ = 2.3 Hz, $J_{4,5}$ = 9.5 Hz, H-4), 3.41 (dd ≈ t, 2H, $J_{5,6'}$ ≈ $J_{6,6'}$ = 10.2 Hz, H-6´), 2.10 (s, 6H, CH_3CO), 1.52 [s, 6H, $(CH_3)_2C$], ppm; **^{13}C NMR** ($CDCl_3$, 100 MHz): *δ* = 170.3 (C, $CH_3\mathit{C}O$), 169.5 (C, O–C=N), 136.8 (C, Ph), 129.1, 128.2, 125.9 (CH, Ph), 103.1 (CH, C-1), 101.5 (CH, PhCH), 75.0 (CH, C-4), 68.9 (CH_2, C-6), 64.1 (CH, C-3), 60.4 (CH, C-2), 59.6 (CH, C-5), 40.1 [C, $(CH_3)_2\mathit{C}$], 23.1 [CH_3, $(\mathit{C}H_3)_2C$], 20.9 (CH_3, $\mathit{C}H_3CO$) ppm; **HRMS** (ESI): calculated for $C_{35}H_{38}N_2O_{12}Na$ 701.2322, found 701.2321 $[M + Na]^+$.

***N,N′*-Bis(ethyl 3,4,6-tri-*O*-acetyl-2-deoxy-1-thio-*β*-D-allopyranosid-2-yl)dimethylmalonamide (15)**

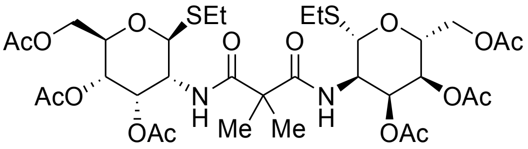

The benzylidene-protected bis(amide) **13** (515 mg, 720 µmol) was dissolved in 60% aqueous acetic acid (10 mL) and the solution stirred at 100 °C for 3 h. The solvent was evaporated and the residue was co-evaporated twice with toluene (10 mL). The crude product was dissolved in pyridine (100 mL) and a catalytic amount of DMAP added. Acetic anhydride (2.05 mL, 2.21 g, 21.6 mmol) was added slowly and the reaction mixture was stirred for 16 h at room temperature (TLC: EtOAc). The solvent was removed under reduced pressure and the residue twice co-evaporated with toluene (100 mL). The residue was purified by flash chromatography on silica gel (eluent: PE/EtOAc 1:1) to yield **15** (440 g, 550 µmol, 77%) as a colourless foam.

$\boldsymbol{R}_f$ 0.61 (EtOAc); $[\alpha]_D^{20}$ = −37.3 (c = 1.10, $CHCl_3$); **^{1}H NMR** ($CDCl_3$, 400 MHz): δ = 6.69 (d, 2H, $J_{2,NH}$ = 8.8 Hz, NH), 5.54 (dd ≈ t, 2H, $J_{2,3} \approx J_{3,4}$ = 2.7 Hz, H-3), 4.91 (dd, 2H, $J_{3,4}$ = 2.7 Hz, $J_{4,5}$ = 10.2 Hz, H-4), 4.69 (d, 2H, $J_{1,2}$ = 10.2 Hz, H-1), 4.30 (ddd ≈ td, 2H, $J_{1,2}$ = 10.2 Hz, $J_{2,3}$ = 3.0 Hz, $J_{2,NH}$ = 8.8 Hz, H-2), 4.25 (dd, 2H, $J_{5,6}$ = 4.7 Hz, $J_{6,6'}$ = 10.2 Hz, H-6), 4.13-4.19 (m, 4H, H-6, H-6′), 3.96 (ddd ≈ td, 2H, $J_{4,5}$ = 10.2, $J_{5,6'}$ = 7.1 Hz, $J_{5,6}$ = 4.0 Hz, H-5), 2.64–2.73 (m, 4H, $SC\mathit{H}_2CH_3$), 2.14 (s, 6H, CH_3CO), 2.04 (s, 6H, CH_3CO), 1.95 (s, 6H, CH_3CO), 1.35 [s, 6H, $(CH_3)_2C$], 1.25 (t, 6H, J = 7.5 Hz, $SCH_2C\mathit{H}_3$) ppm; **^{13}C NMR** ($CDCl_3$, 100 MHz): δ = 172.7 (C, CONH), 170.7, 169.4, 169.1 (C, $CH_3\mathit{C}O$), 81.6 (CH, C-1), 72.6 (CH, C-5), 69.1 (CH, C-3), 66.6 (CH, C-4), 62.6 (CH_2, C-6), 49.8 [C, $(CH_3)_2\mathit{C}$], 49.3 (CH, C-2), 24.1 [CH_3, $(\mathit{C}H_3)_2C$], 23.8 (CH_2, $S\mathit{C}H_2CH_3$), 20.7, 20.6, 20.5 (CH_3, $\mathit{C}H_3CO$), 14.9 (CH_3, $SCH_2\mathit{C}H_3$) ppm; **HRMS** (ESI): calculated for $C_{33}H_{51}N_2O_{16}S_2$ 795.2680, found 795.2692 $[M + H]^+$.

Ac *allo*Box (16)

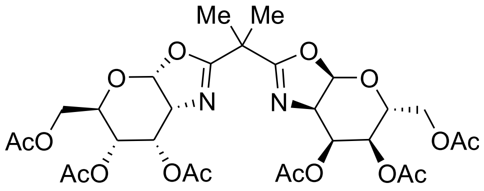

A mixture of the per-acetylated bis(amide) **15** (270 mg, 340 µmol) and MS 4 Å (300 mg) in dry CH_2CH_2 (10 mL) was stirred for 1 h under a nitrogen atmosphere. NIS (184 mg, 820 µmol) was added, and the mixture cooled to −30 °C. Then, TfOH (2.96 µL, 50 µmol) was added, and the mixture stirred for 1 h at −30 °C. The reaction was quenched with Et_3N (200 µL), filtered through Celite®, diluted with CH_2Cl_2, washed successively with saturated aqueous $NaHCO_3$ solution and aqueous sodium thiosulfate solution (3 M), and dried over Na_2SO_4. The solvent was removed under reduced pressure and the residue purified by flash chromatography on silica gel (eluent: EtOAc) to yield **16** (210 mg, 310 µmol, 91%) as a colourless solid.

***R*$_f$** 0.09 (EtOAc); **$[\alpha]_D^{20}$** = +139.5 (*c* = 1.25, $CHCl_3$); **^{1}H NMR** ($CDCl_3$, 400 MHz): δ = 6.01 (d, 2H, $J_{1,2}$ = 7.5 Hz, H-1), 5.28 (dd ≈ t, 2H, $J_{2,3} \approx J_{3,4}$ = 4.7 Hz, H-3), 5.20 (dd, 2H, $J_{3,4} \approx J_{4,5}$ = 5.6 Hz, H-4), 4.24 (dd, 2H, $J_{1,2}$ = 7.5 Hz, $J_{2,3}$ = 4.4 Hz, H-2), 4.15–4.19 (m, 4H, H-6, H-6′), 4.02–4.07 (m, 2H, H-5), 2.09, 2.06, 2.05 (s, 6H, CH_3CO), 1.52 [s, 6H, $(CH_3)_2C$] ppm; **^{13}C NMR** ($CDCl_3$, 100 MHz): δ = 170.5, 170.0, 169.9 (C, $CH_3\mathit{C}O$), 169.3 (C, O–C=N), 101.2 (CH, C-1), 70.1 (CH, C-5), 65.7 (CH, C-4), 65.3 (CH, C-3), 63.7 (CH_2, C-6), 62.7 (CH, C-2), 39.2 [C, $(CH_3)_2\mathit{C}$], 24.1 [CH_3, $(\mathit{C}H_3)_2C$], 20.8, 20.7, 20.6 (CH_3, $\mathit{C}H_3CO$) ppm; **HRMS** (ESI): calculated for $C_{29}H_{39}N_2O_{16}$ 671.2000, found 671.2305 $[M + H]^+$.

Ethyl 4,6-*O*-benzylidene-2-deoxy-3-*O*-[methyl thio(thiocarbonyl)]-2-phthalimido-1-thio-*β*-D-glucopyranoside (17)

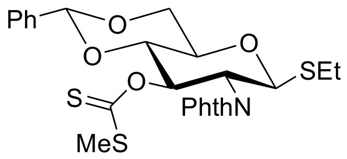

Thioglucoside **7** (1.00 g, 2.27 mmol) was dissolved in dry DMF (15 mL). Sodium hydride (180 mg of 60% dispersion in mineral oil, 4.54 mmol) was added and the reaction mixture stirred for 30 min at room temperature. Carbon disulfide (280 µL, 4.54 mmol) was added and the mixture stirred for an additional 15 min. Subsequently, methyl iodide (280 µL, 4.54 mmol) was added and the stirring continued for 30 min. The reaction mixture was poured onto ice and extracted with EtOAc. The organic phase was washed twice with water, dried over Na_2SO_4 and concentrated. Flash chromatography on silica gel (eluent: PE/EtOAc 3:1) yielded **17** (1.06 g, 1.99 mmol, 88%) as a white foam.

$\boldsymbol{R}_\mathbf{f}$ 0.46 (2:1 PE/EtOAc); $[\alpha]_D^{20}$ = −5.5 (*c* = 1.0, $CHCl_3$); **^{1}H NMR** ($CDCl_3$, 400 MHz): *δ* = 7.82–7.84 (m, 2H, arom.), 7.70–7.73 (m, 2H, arom.), 7.40–7.43 (m, 2H, arom.), 7.31–7.36 (m, 3H, arom.), 6.81 (dd ≈ t, 1H, $J_{2,3} \approx J_{3,4}$ = 9.5 Hz, H-3), 5.64 (d, 1H, $J_{1,2}$ = 10.2 Hz, H-1), 5.55 (s, 1H, CHPh), 4.51 (dd ≈ t, 1H, $J_{1,2} \approx J_{2,3}$ = 10.2 Hz, H-2), 4.43 (dd, 1H, $J_{5,6}$ = 4.1 Hz, $J_{6,6'}$ = 10.2 Hz, H-6), 3.95 (dd ≈ t, 1H, $J_{3,4} \approx J_{4,5}$ = 9.2 Hz, H-4), 3.80–3.97 (m, 2H, H-5, H-6′), 2.63–2.74 (m, 2H, SCH_2CH_3), 2.36 (s, 3H, SCH_3), 1.19 (t, 3H, *J* = 7.5 Hz, SCH_2CH_3) ppm; **^{13}C NMR** ($CDCl_3$, 100 MHz): *δ* = 215.4 (C, OC(SMe)=S), 167.9 (C, NCO), 167.0 (C, NCO), 136.7 (C, arom.), 134.3 (CH, arom.), 134.1 (CH, arom.), 131.7 (C, arom.), 131.2 (C, arom.), 129.0 (CH, arom.), 128.1 (2 CH, arom.), 128.1 (2 CH, arom.), 123.7 (CH, arom.), 123.5 (CH, arom.), 101.4 (CH, PhCH), 81.7 (CH, C-1), 79.5 (CH, C-4), 78.3 (CH, C-3), 70.4 (CH, C-5), 68.6 (CH_2, C-6), 53.8 (CH, C-2), 24.3 (CH_2, SCH_2CH_3), 19.9 (CH_3, SCH_3), 14.8

(CH_3, SCH_2CH_3) ppm; **HRMS** (ESI) calculated for $C_{25}H_{25}NO_6S_3Na$ 554.0742, found 554.0740 $[M + Na]^+$.

Ethyl 4,6-*O*-benzylidene-2,3-dideoxy-2-phthalimido-1-thio-*β*-D-glucopyranoside (18)

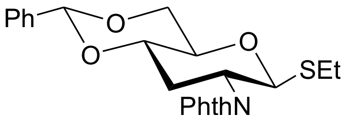

Xanthogenate **17** (500 mg, 940 µmol) was dissolved in dry benzene (10 mL) under a nitrogen atmosphere and Bu_3SnH (470 µL, 1.76 mmol) added. Then AIBN (~20 mg) was added and the reaction mixture refluxed for 15 min. After removal of the solvent the residue was diluted with *n*-hexane and extracted five times with acetonitrile. After removal of the solvent, the residue was purified by flash chromatography on silica gel (eluent: PE/EtOAc 3:1) to yield **18** (380 mg, 900 µmol, 96%) as a white foam. $\boldsymbol{R_f}$ 0.69 (2:1 PE/EtOAc); $[\alpha]_D^{20}$ = −3.8 (*c* = 1.0, $CHCl_3$); **^{1}H NMR** ($CDCl_3$, 400 MHz): *δ* = 7.84–7.86 (m, 2H, arom.), 7.70–7.74 (m, 2H, arom.), 7.45–7.48 (m, 2H, arom.), 7.31–7.38 (m, 3H, arom.), 5.55 (s, 1H, CHPh), 5.41 (d, 1H, $J_{1,2}$ = 10.2 Hz, H-1), 4.42 (ddd, 1H, $J_{1,2}$ = 10.2 Hz, $J_{2,3}$ = 12.2 Hz, $J_{2,3'}$ = 4.4 Hz, H-2), 4.36 (dd, 1H, $J_{5,6}$ = 4.1 Hz, $J_{6,6'}$ = 10.2 Hz, H-6), 3.79 (dd ≈ t, 1H, $J_{5,6'}$ ≈ $J_{6,6'}$ = 10.2 Hz, H-6´), 3.64 – 3.75 (m, 2H, H-4, H-5), 2.60 – 2.75 (m, 3H, H-3, SCH_2CH_3), 2.30 (ddd ≈ td, 1H, $J_{2,3'}$ = 4.4 Hz, $J_{3,3'}$ = 11.6 Hz, $J_{3',4}$ = 4.1 Hz, H-3´), 1.18 (t, 3H, *J* = 7.5 Hz, SCH_2CH_3) ppm; **^{13}C NMR** ($CDCl_3$, 100 MHz): *δ* = 167.9 (C, NCO), 167.2 (C, NCO), 137.2 (C, arom.), 134.2 (2 CH, arom.), 131.7 (C, arom.), 129.1 (CH, arom.), 128.3 (2 CH, arom.), 126.1 (2 CH, arom.), 123.6 (CH, arom.), 123.4 (CH, arom.), 101.7 (CH, PhCH), 83.0 (CH, C-1), 76.6 (CH, C-4), 73.7 (CH, C-5), 69.0 (CH_2, C-6), 49.6 (CH, C-2), 32.7 (CH_2, C-3), 24.3 (CH_2, SCH_2CH_3), 14.9 (CH_3, SCH_2CH_3) ppm; **HRMS** (ESI) calculated for $C_{23}H_{23}NO_5SNa$ 448.1195, found 448.1203 $[M + Na]^+$.

Ethyl 2-amino-4,6-*O*-benzylidene-2,3-dideoxy-1-thio-*β*-D-glucopyranoside (19)

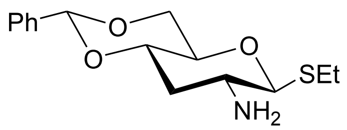

The N-protected 3-deoxy sugar **18** (180 mg, 420 µmol) and ethylene diamine (1.05 mL, 25.40 mmol) were dissolved in absolute ethanol (50 mL) and the resulting mixture refluxed for 2 h (TLC: EtOAc). The solvent was evaporated and the residue co-evaporated twice with toluene. Flash chromatography on silica gel (eluent: EtOAc) yielded **19** (120 mg, 390 µmol, 93%) as a white solid.

$\boldsymbol{R_f}$ 0.28 (EtOAc); $[\alpha]_D^{20}$ = −63.7 (*c* = 1.6, $CHCl_3$); **^{1}H NMR** ($CDCl_3$, 400 MHz): δ = 7.44–7.47 (m, 2H, arom.), 7.31–7.37 (m, 3H, arom.), 5.52 (s, 1H, CHPh), 4.30 (dd, 1H, $J_{5,6}$ = 4.7 Hz, $J_{6,6'}$ = 10.5 Hz, H-6), 4.23 (d, 1H, $J_{1,2}$ = 9.5 Hz, H-1), 3.74 (dd ≈ t, 1H, $J_{5,6'} \approx J_{6,6'}$ = 10.5 Hz, H-6´), 3.63 (ddd, 1H, $J_{3,4}$ = 8.8 Hz, $J_{3',4}$ = 4.1 Hz, $J_{4,5}$ = 9.2 Hz, H-4), 3.41 (ddd ≈ td, 1H, $J_{4,5} \approx J_{5,6'}$ = 10.2 Hz, $J_{5,6}$ = 4.7 Hz, H-5), 2.85–2.91 (m, 1H, $J_{1,2}$ = 9.5 Hz, $J_{2,3}$ = 11.6 Hz, $J_{2,3'}$ = 4.4 Hz, H-2), 2.68–2.77 (m, 2H, S$CH_2$$CH_3$), 2.42 (ddd ≈ td, 1H, $J_{2,3'}$ = 4.4 Hz, $J_{3,3'}$ = 11.9 Hz, $J_{3',4}$ = 4.1 Hz, H-3´), 1.50–1.60 (m, 3H, H-3, NH_2), 1.30 (t, 3H, J = 7.5 Hz, SCH_2CH_3) ppm; **^{13}C NMR** ($CDCl_3$, 100 MHz): δ = 137.2 (C, arom.), 129.0 (CH, arom.), 128.3 (2 CH, arom.), 126.1 (2 CH, arom.), 101.7 (CH, PhCH), 90.9 (CH, C-1), 76.5 (CH, C-4), 73.8 (CH, C-5), 69.0 (CH_2, C-6), 51.6 (CH, C-2), 37.8 (CH_2, C-3), 24.6 (CH_2, SCH_2CH_3), 15.3 (CH_3, SCH_3) ppm; **HRMS** (ESI) calculated for $C_{15}H_{22}NO_3S$ 296.1320, found 296.1323 $[M + H]^+$.

***N,N'*-Bis(ethyl 2-amino-4,6-*O*-benzylidene-2,3-dideoxy-1-thio-*β*-D-glucopyranosid-2-yl)dimethylmalonamide (20)**

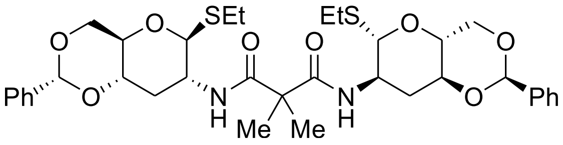

The 3-deoxygenated aminosugar **19** (310 mg, 1.05 mmol) was dissolved under a nitrogen atmosphere in dry CH_2Cl_2 (25 mL) and the solution cooled to 0 °C. Then, Et_3N (280 μL, 2.00 mmol) and dimethylmalonyl dichloride (70 μL, 500 μmol) were added subsequently. After approximately 2 h (monitored by TLC: EtOAc), the solvent was removed *in vacuo*, and the product purified by flash chromatography on silica gel (eluent: PE/EtOAc 1:1) to yield **20** (340 mg, 500 μmol, quant.) as a white solid.

$\boldsymbol{R_f}$ 0.50 (1:1 PE/EtOAc); $[\alpha]_D^{20}$ = −76.8 (*c* = 1.0, $CHCl_3$); ^{1}**H NMR** ($CDCl_3$, 400 MHz): δ = 7.42–7.45 (m, 4H, arom.), 7.31–7.36 (m, 6H, arom.), 6.58 (d, 2H, $J_{2,NH}$ = 8.8 Hz, NH), 5.50 (s, 1H, CHPh), 4.48 (d, 2H, $J_{1,2}$ = 10.2 Hz, H-1), 4.30 (dd, 2H, $J_{5,6}$ = 4.7 Hz, $J_{6,6'}$ = 10.5 Hz, H-6), 4.09 (ddd, 2H, $J_{1,2}$ = 10.2 Hz, $J_{2,3}$ = 11.6 Hz, $J_{2,3'}$ = 4.4 Hz, H-2), 3.74 (dd ≈ t, 2H, $J_{5,6'}$ ≈ $J_{6,6'}$ = 10.2 Hz, H-6′), 3.62 (ddd, 2H, $J_{3,4}$ = 8.8 Hz, $J_{3',4}$ = 4.0 Hz, $J_{4,5}$ = 9.2 Hz, H-4), 3.41 (ddd ≈ td, 2H, $J_{4,5}$ ≈ $J_{5,6'}$ = 9.2 Hz, $J_{5,6}$ = 4.7 Hz, H-5), 2.66–2.75 (m, 4H, SCH_2CH_3), 2.47 (ddd ≈ td, 2H, $J_{2,3'}$ = 4.4 Hz, $J_{3,3'}$ = 11.9 Hz, $J_{3',4}$ = 4.0 Hz, H-3′), 1.66–1.75 (m, 2H, $J_{2,3}$ = 11.6 Hz, $J_{3,3'}$ = 11.9 Hz, $J_{3,4}$ = 8.8 Hz, H-3), 1.45 (s, 6H, CH_3), 1.25 (t, 6H, *J* = 7.5 Hz, SCH_2CH_3) ppm; ^{13}**C NMR** ($CDCl_3$, 100 MHz): δ = 172.8 (C, CONH), 137.1 (C, Ph), 129.0, 128.2, 126.0 (CH, Ph), 101.5 (CH, PhCH), 86.2 (CH, C-1), 75.6 (CH, C-4), 74.0 (CH, C-5), 68.8 (CH_2, C-6), 49.6 (CH, C-2), 48.6 [C, $(CH_3)_2C$)], 35.8 (CH_2, C-3), 23.9 (CH_2, SCH_2CH_3), 23.8 [CH_3, $(CH_3)_2C$], 14.9 (CH_3, SCH_3) ppm; **HRMS** (ESI) calculated for $C_{35}H_{46}N_2O_8S_2Na$ 709.2593, found 709.2610 $[M + Na]^+$.

3-*O*-Deoxy *gluco*Box (21)

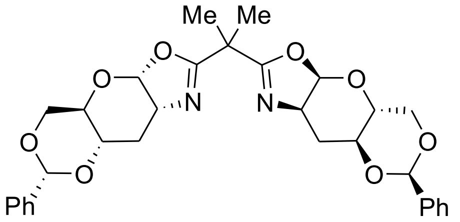

A mixture of the benzylidene-protected bis(amide) **20** (150 mg, 220 μmol) and MS 4 Å (150 mg) in dry CH_2CH_2 (5 mL) was stirred for 1 h under a nitrogen atmosphere. NIS (118 mg, 520 μmol) was added, and the mixture cooled to −30 °C. Then, TfOH (3 μL, 30 μmol) was added, and the reaction mixture stirred for 1 h at −30 °C. The reaction was quenched with Et_3N (100 μL), filtered through Celite®, diluted with CH_2Cl_2, washed successively with saturated aqueous $NaHCO_3$ solution and aqueous sodium thiosulfate solution (3 M), and dried over Na_2SO_4. The solvent was removed under reduced pressure and the residue purified by flash chromatography on silica gel (eluent: EtOAc) to yield **21** (109 mg, 190 μmol, 89%) as a white solid.

***R*$_f$** 0.17 (EtOAc); **$[\alpha]_D^{20}$** = +160.6 (*c* = 0.98, $CHCl_3$); **^{1}H NMR** ($CDCl_3$, 400 MHz): *δ* = 7.44–7.48 (m, 4H, arom.), 7.30–7.37 (m, 6H, arom.), 5.86 (d, 2H, $J_{1,2}$ = 7.1 Hz, H-1), 5.54 (s, 2H, CHPh), 4.35 (dd, 2H, $J_{5,6}$ = 4.0 Hz, $J_{6,6'}$ = 9.9 Hz, H-6), 4.08–4.14 (m, 2H, H-2), 3.62–3.75 (m, 6H, H-4, H-5, H-6′), 2.63 (ddd, 2H, $J_{2,3'}$ = 7.5 Hz, $J_{3,3'}$ = 13.3 Hz, $J_{3',4}$ = 4.4 Hz, H-3′), 1.63 (ddd, 2H, $J_{2,3}$ = 8.8 Hz, $J_{3,3'}$ = 13.3 Hz, $J_{3,4}$ = 7.8 Hz, H-3), 1.45 (s, 6H, CH_3) ppm; **^{13}C NMR** ($CDCl_3$, 100 MHz): *δ* = 168.2 (C, O-C=N), 137.1 (C, arom.), 129.0 (CH, arom.), 128.2 (2 CH, arom.), 126.1 (2 CH, arom.), 102.6 (CH, C-1), 101.6 (CH, PhCH), 73.7 (CH, C-4), 68.8 (CH_2, C-6), 65.8 (CH, C-5), 59.3 (CH, C-2), 39.1 [C, $(CH_3)_2C$)], 32.4 (CH_2, C-3), 23.2 [CH_3, $(CH_3)_2C$] ppm; **HRMS** (ESI) calculated for $C_{31}H_{35}N_2O_8$ 563.2393, found 563.2385 $[M + H]^+$.

***N,N'*-Bis(ethyl 4,6-di-*O*-acetyl-2-amino-2,3-dideoxy-1-thio-*β*-D-glucopyranosid-2-yl)dimethylmalonamide (22)**

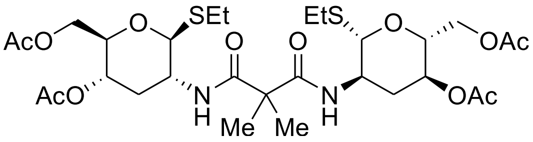

The benzylidene-protected bis(amide) **20** (1.17 g, 1.70 mmol) was dissolved in acetic acid (25 mL, 60%) and the solution stirred at 100 °C for 3 h. The solvent was evaporated and the residue twice co-evaporated with toluene (25 mL).

The crude product was dissolved in pyridine (150 mL) and a catalytic amount of DMAP added. Acetic anhydride (3.21 mL, 3.47 g, 34.00 mmol) was added slowly and the reaction mixture stirred for 5 h at room temperature (TLC: PE/EtOAc 1:2). The solvent was removed under reduced pressure and the residue twice co-evaporated with toluene (200 mL). The residue was purified by flash chromatography on silica gel (eluent: PE/EtOAc 1:2) to yield **22** (1.07 g, 1.58 mmol, 93%) as a colourless foam.

$\boldsymbol{R_f}$ 0.28 (1:2 PE/EtOAc); $[\alpha]_D^{20}$ = −13.2 (c = 1.0, $CHCl_3$); **^{1}H NMR** ($CDCl_3$, 400 MHz): δ = 6.57 (d, 2H, $J_{2,NH}$ = 7.5 Hz, NH), 4.76 (ddd ≈ dt, 2H, $J_{3,4} \approx J_{4,5}$ = 10.5 Hz, $J_{3',4}$ = 4.7 Hz, H-4), 4.45 (d, 2H, $J_{1,2}$ = 9.9 Hz, H-1), 4.14-4.17 (m, 4H, H-6, H-6´), 3.91-4.01 (m, 2H, H-2), 3.60 (m, 2H, H-5), 2.61-2.71 (m, 4H, SCH_2CH_3), 2.48-2.53 (m, 2H, H-3´), 2.03 (s, 6H, CH_3CO), 2.00 (s, 6H, CH_3CO), 1.54-158 (m, 2H, H-3), 1.39 [s, 6H, $(CH_3)_2C$], 1.23 (t, 6H, J = 7.5 Hz, SCH_2CH_3) ppm; **^{13}C NMR** ($CDCl_3$, 100 MHz): δ = 172.8 (C, CONH), 170.7, 169.5 (C, CH_3CO), 85.5 (CH, C-1), 77.9 (CH, C-5), 66.3 (CH, C-4), 62.8 (CH_2, C-6), 49.5 [C, $(CH_3)_2C$)], 48.2 (CH, C-2), 35.4 (CH_2, C-3), 24.0 (CH_2, SCH_2CH_3), 23.9 [CH_3, $(CH_3)_2C$], 20.8, 20.7 (CH_3, CH_3CO), 14.9 (CH_3, SCH_3) ppm; **HRMS** (ESI) calculated for $C_{29}H_{47}N_2O_{12}S_2$ 679.2570, found 679.2597 $[M + H]^+$.

Ac 3-*O*-Deoxy *gluco*Box (23)

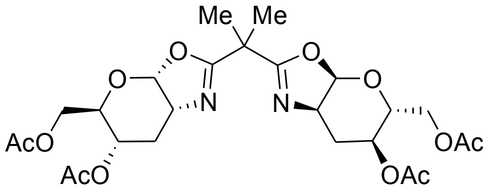

A mixture of the 3-deoxygenated, per-acetylated bis(amide) **22** (500 mg, 740 μmol) and MS 4 Å (500 mg) in dry CH_2CH_2 (20 mL) was stirred for 1 h under a nitrogen atmosphere. NIS (400 mg, 1.78 mmol) was added, and the mixture cooled to −30 °C. Then, TfOH (15 μL, 30 μmol) was added, and the reaction mixture stirred for 1 h at −30 °C. The reaction was quenched with Et_3N (500 μL), filtered through Celite®, diluted with CH_2Cl_2, washed successively with saturated aqueous $NaHCO_3$ solution and aqueous sodium thiosulfate solution (3 M), and dried over Na_2SO_4. The solvent was removed under reduced pressure and the residue purified by flash chromatography on silica gel (eluent: EtOAc) to yield **23** (360 mg, 650 μmol, 88%) as a white solid.

***R*$_f$** 0.04 (EtOAc); **[*α*]$_D^{20}$** = +82.3 (*c* = 1.23, $CHCl_3$); **^{1}H NMR** ($CDCl_3$, 400 MHz): *δ* = 5.90 (d, 2H, $J_{1,2}$ = 7.6 Hz, H-1), 4.95 (m, 2H, H-4), 4.16 (dd, 2H, $J_{5,6}$ = 3.0, $J_{6,6'}$ = 12.1, H-6), 4.11–4.13 (m, 2H, H-2), 4.07 (dd, 2H, $J_{5,6'}$ = 5.3, $J_{6,6'}$ = 12.1, H-6′), 3.78 (ddd ≈ dt, 2H, $J_{4,5}$ = 7.9, $J_{5,6}$ = 3.0, $J_{5,6'}$ = 5.3, H-5), 2.20-2.29 (m, 2H, H-3′), 2.07 (ddd, 2H, $J_{2,3}$ = 6.6 Hz, $J_{3,3'}$ = 15.8 Hz, $J_{3,4}$ = 5.1 Hz, H-3), 2.01 (s, 6H, CH_3CO), 1.99 (s, 6H, CH_3CO), 1.54 [s, 6H, $(CH_3)_2C$] ppm; **^{13}C NMR** ($CDCl_3$, 100 MHz): *δ* = 170.5, 170.0 (C, CH_3*C*O), 168.4 (C, O-C=N), 100.5 (CH, C-1), 68.4 (CH, C-5), 65.5 (CH, C-4), 63.8 (CH_2, C-6), 60.0 (CH, C-2), 38.8 [C, $(CH_3)_2$*C*)], 26.8 (CH_2, C-3), 23.9 [CH_3, (*C*H_3)$_2$C], 21.1, 20.6 (CH_3, *C*H_3CO) ppm; **HRMS** (ESI) calculated for $C_{25}H_{35}N_2O_{12}$ 555.2190, found 555.2188 $[M + H]^+$.

***N,N′*-Bis(ethyl 3-*O*-formyl-4,6-*O*-benzylidene-2-deoxy-1-thio-*β*-D-glucopyranosid-2-yl)dimethylmalonamide (25)**

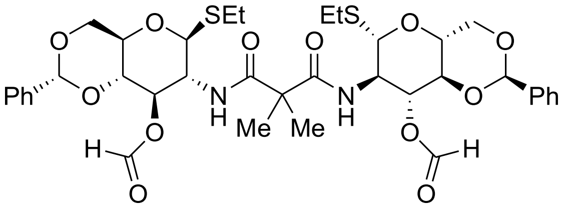

The acetoformic anhydride necessary for the 3-*O*-formylation was prepared by stirring 1 eq. of acetic anhydride and 1.5 eq. of formic acid at 60 °C for 2 h. Then, the *gluco*-configured bis(amide) **24** (250 mg, 260 µmol) was dissolved in pyridine (15 mL) and a catalytic amount of DMAP added. The reaction mixture was cooled to –20 °C and acetoformic anhydride (310 mg, 3.50 mmol) added dropwise. The reaction mixture was subsequently stirred for 1 h at –20 °C (TLC: PE/EtOAc 1:1). The solvent was removed under reduced pressure and the residue twice co-evaporated with toluene (20 mL). The residue was purified by flash chromatography on silica gel (eluent: PE/EtOAc 2:1) to yield **25** (264 mg, 340 µmol, 98%) as a colour-less foam.

$\boldsymbol{R_f}$ 0.53 (1:1 PE/EtOAc); $[\alpha]_D^{20}$ = −91.6 (*c* = 1.16, $CHCl_3$); **^{1}H NMR** ($CDCl_3$, 400 MHz): δ = 8.12 (s, 2H, OC(O)*H*), 7.42–7.44 (m, 4H, Ph), 7.31-7.36 (m, 6H, Ph), 6.69 (d, 2H, $J_{2,NH}$ = 9.2 Hz, NH), 5.50 (s, 2H, CHPh), 5.46 (dd ≈ t, 2H, $J_{2,3} \approx J_{3,4}$ = 9.56 Hz, H-3), 4.77 (d, 2H, $J_{1,2}$ = 10.2 Hz, H-1), 4.33 (dd, 2H, $J_{5,6}$ = 5.1 Hz, $J_{6,6'}$ = 10.2 Hz, H-6), 4.17 (dd, 2H, $J_{1,2}$ = 10.2 Hz, $J_{2,3}$ = 9.5 Hz, H-2), 3.73-3.79 (m, 4H, H-4, H-6′), 3.58 (ddd ≈ dt, 2H, $J_{4,5} \approx J_{5,6'}$ = 9.9 Hz, $J_{5,6}$ = 5.1 Hz, H-5), 2.62–2.76 (m, 4H, SCH_2CH_3), 1.38 [s, 6H, $(CH_3)_2C$], 1.24 (t, 6H, *J* = 7.5 Hz, SCH_2CH_3) ppm; **^{13}C NMR** ($CDCl_3$, 100 MHz): δ = 173.4 (C, CONH), 161.4 (CH, OC(O)H), 136.6 (C, Ph), 129.1, 128.2, 126.1 (CH, Ph), 101.5 (CH, PhCH), 84.2 (CH, C-1), 78.3 (CH, C-4), 73.1 (CH, C-3), 70.4 (CH, C-5), 68.4 (CH_2, C-6), 53.4 (CH, C-2), 50.6 [C, $(CH_3)_2C$], 24.2 [CH_3, $(CH_3)_2C$], 24.0

(CH_2, SCH_2CH_3), 14.8 (CH_3, SCH_2CH_3) ppm; **HRMS** (ESI): calculated for $C_{37}H_{47}N_2O_{12}NaS_2$ 775.2570, found 775.2579 $[M + H]^+$.

3-*O*-Formyl *gluco*Box (26)

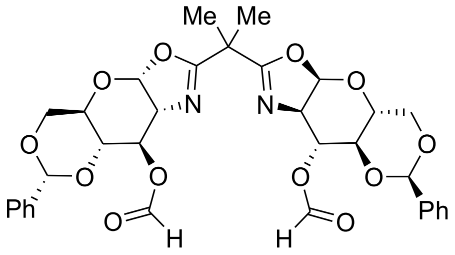

A mixture of the 3-O-formylated bis(amide) **25** (410 mg, 530 µmol) and MS 4 Å (400 mg) in dry CH_2CH_2 (20 mL) was stirred for 1 h under a nitrogen atmosphere. NIS (290 mg, 1.27 mmol) was added, and the mixture cooled to −30 °C. Then, TfOH (6.5 µL, 70 µmol) was added, and the mixture stirred for 1 h at −30 °C. The reaction was quenched with Et_3N (400 µL), filtered through Celite®, diluted with CH_2Cl_2, washed successively with saturated aqueous $NaHCO_3$ solution and aqueous sodium thio-sulfate solution (3 M), and dried over Na_2SO_4. The solvent was removed under reduced pressure and the residue purified by flash chromatography on silica gel (eluent: EtOAc) to yield **26** (290 mg, 456 µmol, 85%) as a colourless solid.

$\boldsymbol{R_f}$ 0.29 (EtOAc); $[\alpha]_D^{20}$ = −3.49 (*c* = 0.93, $CHCl_3$); **^{1}H NMR** ($CDCl_3$, 400 MHz): δ = 8.12 (s, 2H, OC(O)*H*), 7.42–7.46 (m, 4H, Ph), 7.32–7.36 (m, 6H, Ph), 6.01 (d, 2H, $J_{1,2}$ = 7.5 Hz, H-1), 5.52 (s, 2H, CHPh), 5.26 (dd, 2H, $J_{2,3}$ = 3.4 Hz, $J_{3,4}$ = 7.1 Hz, H-3), 4.38 (dd, 2H, $J_{5,6}$ = 4.7 Hz, $J_{6,6'}$ = 10.2 Hz, H-6), 4.16 (dd, 2H, $J_{1,2}$ = 7.5 Hz, $J_{2,3}$ = 3.4 Hz, H-2), 3.73–3.84 (m, 4H, H-4, H-5), 3.65 (dd ≈ t, 2H, $J_{5,6'} \approx J_{6,6'}$ = 9.9 Hz, H-6´), 1.53 [s, 6H, $(CH_3)_2C$] ppm; **^{13}C NMR** ($CDCl_3$, 100 MHz): δ = 169.7 (C, O–C=N), 160.0 (CH, OC(O)H), 136.6 (C, Ph), 129.1, 128.2, 126.0 (CH, Ph), 101.9 (CH, PhCH), 101.5 (CH, C-1), 77.8 (CH, C-4), 74.1 (CH, C-3), 68.4 (CH_2, C-6), 67.2 (CH, C-2),

63.0 (CH, C-5), 39.2 [C, $(CH_3)_2C$], 23.2 [CH_3, $(CH_3)_2C$] ppm; **HRMS** (ESI): calculated for $C_{33}H_{35}N_2O_{12}$ 651.2190, found 701.651.2178 $[M + H]^+$.

Determination of enantiomeric excesses by gas chromatography on a chiral stationary phase: A racemic sample of the product was analysed by GC on the chiral stationary phase to obtain the retention times of both enantiomers. Then an enantiomerically enriched sample was injected and the enantiomeric excess was determined from the resulting chromatogram by peak integration.

Analytical data for the cyclopropanation products

Absolute configurations were assigned by the sign of the optical rotation of the respective compound and comparison with literature data.

Ethyl (1*S*,2*S*)-2-phenylcyclopropanecarboxylate (*trans* **6**) [1]

$[\alpha]_D^{20}$ = +223 (c = 1.0, $CHCl_3$); ^{1}**H NMR** ($CDCl_3$, 400 MHz): δ = 7.07–7.28 (m, 5H, Ph), 4.15 (q, 2H, J = 7.1 Hz, OCH_2CH_3), 2.50 (ddd, 1H, J = 4.2, 6.5, 9.3 Hz, PhCH), 1.89 (ddd, 1H, J = 4.2, 5.3, 8.4 Hz, $CHCO_2Et$), 1.58 (ddd, 1H, J = 4.6, 5.3, 9.3 Hz, CH_2), 1.29 (ddd, 1H, J = 4.7, 6.2, 8.6 Hz, CH_2), 1.27 (3H, t, J = 7.1 Hz, OCH_2CH_3) ppm; ^{13}**C NMR** ($CDCl_3$, 100 MHz): δ = 173.3 (C, $CHCO_2Et$), 140.0 (C, Ph), 128.4, 126.4, 126.0 (CH, Ph), 160.6 (CH_2, OCH_2CH_3), 26.1 (CH, CHPh), 24.1 (CH, $CHCO_2Et$), 17.0 (CH_2, CH_2), 4.2 (CH_3, OCH_2CH_3) ppm; **MS** (EI): calculated for $C_{12}H_{14}O_2$ 190.0994, found 190.0980 $[M]^+$.

Retention times (GC):

racemic mixture: t_R = 67.45 min, t_R = 68.28 min

product: t_R = 67.75 min (minor), t_R = 68.36 min (major).

Ethyl (1*S*,2*R*)-2-phenylcyclopropanecarboxylate (*cis* **6**) [1]

$[\alpha]_D^{20}$ = +26 (*c* = 1.0, $CHCl_3$); **^{1}H NMR** ($CDCl_3$, 400 MHz): *δ* = 7.17–7.26 (m, 5H, Ph), 3.86 (q, 2H, *J* = 7.2 Hz, OCH_2CH_3), 2.54–2.61 (m, 1H, PhCH), 2.07 (ddd, 1H, *J* = 5.6, 7.8, 9.3 Hz, $CHCO_2Et$), 1.70 (ddd, 1H, *J* = 5.2, 5.5, 7.5 Hz, CH_2), 1.26 (ddd, 1H, *J* = 5.1, 7.9, 8.6 Hz, CH_2), 0.96 (t, 3H, *J* = 7.1 Hz, OCH_2CH_3) ppm; **^{13}C NMR ($CDCl_3$, 100 MHz,):** *δ* = 171.0 (C, $CHCO_2Et$), 136.5 (C, Ph), 129.3, 127.8, 126.6 (CH, Ph), 60.1 (CH_2, OCH_2CH_3), 25.4 (CH, PhCH), 21.8 (CH, $CHCO_2Et$), 14.0 (CH_3, OCH_2CH_3), 11.1 (CH_2, CH_2) ppm; **MS** (EI): calculated for $C_{12}H_{14}O_2$ 190.0994, found 190.0980 $[M]^+$.

Retention times (GC):

racemic mixture: t_R = 63.53 min, t_R = 65.67 min

product: t_R = 63.62 min (major), t_R = 65.89 min (minor).

References

1. Evans, D. A.; Woerpel, K. A.; Hinman, M. M.; Faul, M. M. *J. Am. Chem. Soc.* **1991**, *113,* 726–728. doi:10.1021/ja00002a080
